# miR-663a inhibits tumor growth and invasion by regulating TGF-β1 in hepatocellular carcinoma

**DOI:** 10.1186/s12885-018-5016-z

**Published:** 2018-11-28

**Authors:** Chengshuo Zhang, Baomin Chen, Ao Jiao, Feng Li, Ning Sun, Guoqing Zhang, Jialin Zhang

**Affiliations:** 1 Hepatobiliary Surgery Department and Unit of Organ Transplantation, The First Hospital of China Medical University, 155#, Nanjingbei street, Heping district, Shenyang, Liaoning People’s Republic of China; 2 Hepatobiliary Surgery Department, The First Affiliated Hospital, Sun Yat-sen University, Guangzhou, 510000 People’s Republic of China; 3 Hepatobiliary Surgery Department, The First Affiliated Hospital of Zhengzhou University, Zhengzhou, 450052 People’s Republic of China

**Keywords:** Hepatocellular carcinoma (HCC), miR-663a, Transforming growth factor β1 (TGF-β1), Proliferation, Invasion

## Abstract

**Background:**

The dysregulation of miR-663a is frequently observed in many human cancers. However, the functional role and precise mechanism of miR-663a have been controversial in hepatocellular carcinoma (HCC) and need to be studied in depth.

**Methods:**

The expression of miR-663a was detected in human cell lines and HCC tissues by quantitative RT-PCR (qRT-PCR), and data from the Cancer Genome Atlas (TCGA). Cell proliferation was investigated using MTS, EdU, colony formation assays, and xenograft animal experiments, and the cell invasion capacity was evaluated using the transwell assay. The target gene of miR-663a was identified by qRT-PCR, Western blot, and dual-luciferase reporter assays. The clinicopathological features of miR-663a and the correlation between miR-663a and TGF-β1 expression were also investigated in the clinical samples of HCC.

**Results:**

miR-663a was significantly downregulated in HCC cells relative to immortal normal liver cells, as indicated using qRT-PCR, and the lower expression of miR-663a was also confirmed in HCC tissue samples and the data from TCGA. The expression of miR-663a in HCC tissue samples was statistically significantly associated with size and the number of tumors. In addition, the upregulation of miR-663a inhibited the proliferation and invasion of HCC cells in vitro. Further study showed that miR-663a directly targeted transforming growth factor beta 1 (TGF-β1) to suppress HCC invasion, and that the inhibitory effect of miR-663a on cell invasion could be regulated by TGF-β1. In vivo studies showed that miR-663a significantly inhibited tumor growth. A negative correlation between miR-663a and TGF-β1 expression was also confirmed from the clinical samples of HCC.

**Conclusions:**

miR-663a acts as a tumor suppressor and exerts a substantial role in inhibiting the proliferation, invasion, and tumorigenesis of HCC by regulating TGF-β1 in vitro and in vivo. These observations indicate that miR-663a may be a suitable diagnostic, therapeutic, and prognostic target for the treatment of HCC.

**Electronic supplementary material:**

The online version of this article (10.1186/s12885-018-5016-z) contains supplementary material, which is available to authorized users.

## Background

Primary liver cancer is estimated to be the fourth most common malignant tumor and the third leading cause of cancer-related death in China [1]. Hepatocellular carcinoma (HCC) accounts for more than 90% of cases of primary liver cancer and has a global mortality rate of 94% [2]. The prognosis of HCC patients remains unsatisfactory despite the surgical resection, liver transplantation and ablation therapy available [3]. Therefore, further research into new molecular markers for diagnosis and the discovery of vital target for genetic therapy are of great clinical significance to the improvement of the comprehensive effect of HCC.

In the case of HCC, cancer development involves the interaction of several multistep genetic and epigenetic processes. MicroRNAs (miRNAs) are a class of epigenetically regulated small non-coding RNAs that play a vital role in modulating gene expressions post-transcriptionally by the translational repression or degradation of target mRNAs [4]. It has been shown that the abnormal expression of miRNAs can function as oncogenes or tumor suppressors, which has already been reported in a number of cancers, including lung cancer [5], ovarian cancer [6], gastric cancer [7], colorectal cancer [8], liver cancer [9], and breast cancer [10], showing that there may be a close relationship between miRNAs and the several biological processes such as tumor initiation, progression, metastasis, and drug resistance. However, the molecular mechanism by which miRNA are involved in HCC is still unclear and needs to be elucidated.

miR-663a is located in the chromosome 20q11.1 and has been reported to be closely related with the biological behavior of cell differentiation, inflammation, autoimmune diseases, and cancer [11–14]. However, the function of miR-663a in tumor development has not yet been clearly elucidated. It has been revealed that miR-663a can function either as tumor suppressors or oncogenes that play an important role in the progression of many tumors [15–21]. Remarkably, the role of miR-663a in HCC has remained controversial [22, 23].

Herein, we identified that miR-663a was downregulated in the HCC tissues and cells, and was inversely correlated with the clinicopathological features of tumor size and number in HCC patients. In addition, miR-663a acted as a tumor suppressor by inhibiting cell proliferation, invasion, and tumorigenesis, both in vitro and in vivo. Furthermore, miR-663a targeted TGF-β1 directly to inhibit cell invasion, and the negative correlation between the expression of miR-663a and TGF-β1 was also determined in human HCC tissues. The results of this study may clearly verify the regulatory mechanism of miR-663a and provide beneficial target and approach for the clinical diagnosis, therapy and prognosis of HCC in the future.

## Methods

### Patient information and tissue specimens

A total of 186 liquid nitrogen-frozen tumor specimens and another 60 in paired carcinoma and para-carcinoma tissues from patients with HCC who underwent hepatectomy were obtained from Hepatobiliary Surgery Department and Unit of Organ Transplantation, the First Hospital of China Medical University (Shenyang, China) between Jan, 2013 and Feb, 2016. All the samples from the patients were histologically confirmed and classified according to the 7th TNM staging system of the AJCC Cancer Staging Manual, and none of the patients had the history of other malignancy or received preoperative chemotherapy, radiotherapy or radiofrequency ablation therapy.

### TCGA data analysis

The Cancer Genome Atlas (TCGA) open access dataset (http://cancergenome.nih.gov/) was used to obtain miRNA expression of human HCC by Dec 30th,2015. Normalized TCGA level-3 miRNA-seq data (reads per kilobase per million mapped reads-RPKM) was compiled using R project (https://www.r-project.org). Among a total of 426 hybridization data,45 patients had the miR-663a expression data in HCC tissues and its non-tumor paired tissues and none of the patients had the history of other malignancy or received preoperative chemotherapy, radiotherapy or radiofrequency ablation therapy.

### Cell culture

The Huh-7, HCC-LM3, SK-HEP1, MHCC-97H, MHCC-97 L and L02 human cell lines were obtained from the National Infrastructure of Cell Line Resource (http://www.cellresource.cn. Beijing, China) in 2014 and 293 T cell line was purchased from American Type Culture Collection (ATCC, Manassas, VA, USA) in 2014. The cells lines have been tested for mycoplasma contamination every 3 months with the MycoProbe Mycoplasma Detection Kit (R&D Systems, China) and they were free from mycoplasma contamination. SK-HEP1 and L02 were grown in RPMI 1640 medium (KeyGEN, China) supplemented with 100 U/mL penicillin and 100 µg/mL streptomycin in the presence of 10% fetal bovine serum (Gibco, USA) and incubated in a humidified atmosphere containing 5% CO_2_ at 37 °C. Huh-7, HCC-LM3, MHCC-97H, MHCC-97 L and 293 T were maintained in Dulbecco’s modified Eagle’s medium (High glucose) (Hyclone, USA) supplemented with 100 U/mL penicillin and 100 µg/mL streptomycin in the presence of 10% fetal bovine serum (Gibco, USA) and incubated in a humidified atmosphere containing 5% CO_2_ at 37 °C.

### RNA extraction and real-time PCR

Total RNA including the small RNA was extracted from tissue samples and cells by RNAiso plus (Takara, China) according to the manufacturer’s protocol. The purity and the concentration of the RNA was assessed with a NanoDrop 1000 spectrophotometer (Thermo Scientific, USA) at the wavelengths of 260 and 280 nm. Only the RNA sample with a ratio of A260/A280 = 1.8~ 2.0 was considered appropriate for further experiment. For analysis of miRNA, mature miRNA was synthesized from total RNA using a Mir-X miRNA First-Strand Synthesis Kit (Clontech, China). Quantitative Real time PCR (qRT-PCR) was performed on ABI7500 Real Time System with SYBR Premix Ex Taq™ II (Tli RNaseH Plus) (Takara, China). The expression of the target miRNA was normalized to that of the internal control, U6. For quantification of targeted mRNA, cDNA was generated using PrimeScript RT Master Mix (TaKaRa, China), and qRT-PCR was performed on a TP800 Real Time System with SYBR Premix Ex Taq™ II (Tli RNaseH Plus) (Takara, China). Target mRNA expression was normalized against GAPDH. The fold changes between groups were determined using the comparative Ct method (2 − ^ΔΔCT^). The qRT-PCR primers for miR-663a were designed from Tiagen (Beijing, China), U6 was obtained by Clontech (Dalian, China), mRNA primers were purchased from Sangon biotech (Shanghai, China). The primer sequences are listed in Table 1.

**Table 1:** Primers used for quantitative real-time PCR analysis

Primer	Sequence (5′-3′)	
miR-663a	Forward	CTCGCTTCGGCAGCACA
	Reverse	AACGCTTCACGAATTTGCGT
U6	Forward	CTCAACTGGTGTCGTGGAGTCGGCAATTCAGTTGAGGCGGTCCC
	Reverse	ACACTCCAGCTGGGAGGCGGGCGCCGCGG
TGF-β1	Forward	CGACTCGCCAGAGTGGTTAT
	Reverse	AGTGAACCCGTTGATGTCCA
GAPDH	Forward	CAGGAGGCATTGCTGATGAT
	Reverse	GAAGGCTGGGGCTCATTT

### Oligonucleotides transfection

The miR-663a agomiR, antagomiR, small interfering RNA for TGF-β1 (si TGF-β1) and their respective control, purchased from Genepharma Inc. (Suzhou, China), were used to transfect HCC cells by using Lipofectamine 3000 (Invitrogen, Carlsbad, CA) according to the manufacturer’s protocol. Cells were incubated 48 h prior to harvest for all assays. For MTS, EdU and colony formation assays, cells were plated 24 h following RNA transfection. The sequences of oligonucleotides were used as follows: miR-663a agomiR, sense 5′-AGGCGGGGCGCCGCGGGACCGC-3′ and antisense 5′-GGUCCCGCGGCGCCCCGCCUUU-3′; miRNA negative control, sense 5′-UUCUCCGAACGUGUCACGUTT-3′ and antisense 5′-ACGUGACACGUUCGGAGAATT-3′; miR-663a antagomiR, 5′- GCGGUCCCGCGGCGCCCCGCCU-3′; negative control, 5′- UUGUACUACACAAAAGUACUG-3′; si-TGF-β1, 5′- CCCACAACGAAAUCUAUGATT -3′.

### Luciferase assay

The wild-type TGF-β1–3′UTR-WT and mutant-type TGF-β1–3′UTR-MUT containing the putative binding site of miR-663a were established and cloned in the firefly luciferase expressing vector pmiR-REPORT (ABI, Foster, USA).293 T cells were seeded into 24-well plates, and co-transfected with either pmiR-TGF-β1–3′UTR-WT or the pmiR-TGF-β1–3′UTR-MUT, together with the renilla luciferase-expressing vector (pRL, Promega) and miR-663a agomiR, miR-663a antagomiR or respective control using Lipofectamine 3000 (Invitrogen, Carlsbad, USA). Cells were collected 48 h after transfection, and the relative activities were calculated by normalizing the firefly luciferase to the renilla luciferase using the Dual-Luciferase Reporter Assay System (Promega, Madison, WI) according to the instructions.

### MTS assay

The effect of miR-663a on HCC cell viability was measured using the CellTiter 96 Aqueous One Solution cell proliferation assay (Promega, Madison, WI, USA) according to the manufacturer’s instruction. Briefly, cells were seeded into 96-well plates at a concentration of 1 × 10^3^ cells/well to adhere overnight. The cells were then transfected and maintained as indicated above. Every 24 h, 20 μl one solution reagent was added to each well, the absorbance value at 490 nm was measured using an ELISA reader (BioTek, VT, USA) after the mixture were incubated for 3 h at 37 °C. The viability ratio was calculated according to the following formula: Viability ratio = [(absorbance of experimental group − absorbance of blank group)/ (absorbance of control group − absorbance of blank group)] × 100%.

### 5-ethynyl-2′-deoxyuridine (EdU) incorporation assay

Proliferating cells were stained with EdU using the Cell-Light EdU DNA Cell Proliferation Kit (RIBOBio Co., Guangzhou, China). Briefly, 5 × 10^3^ cells/well were seeded into 96-well plates, and then transfected and maintained as indicated above. After 48 h, 50 μmol/L of EdU was added into the cells for 4 h at 37 °C. After fixation with 4% (*w*/*v*) paraformaldehyde for 30 min, the cells were treated with 0.5% (*v*/v) Triton X-100 for 20 min and rinsed with PBS three times. Thereafter, the cells were exposed to 100 μL of 1 × Apollo® reaction cocktail for 30 min and incubated with 5 μg/mL of Hoechst 33342 to stain the cell nuclei for 30 min. EdU-labeled cells and Hoechst 33342-stained cells were counted in 10 random fields of view using a fluorescent microscope (Olympus IX71, Tokyo, Japan). The percentage of EdU-positive cells was calculated as the number of EdU-positive cells/the number of Hoechst-positive cells.

### Colony formation assay

Five hundred cells were placed in six-well plates, and then transfected and maintained in complete medium for 2 weeks. Colonies were fixed with 4% paraformaldehyde, stained with 0.1% crystal violet and counted.

### Invasion assay

Invasion experiment was performed using 24-well plates transwell chamber (Corning Costar, USA) according to the manufacturer’s instructions. After 48-h post-transfection, 2 × 10^5^ cells were resuspended in serum-free medium and added into the 50 μL matrigel-coated upper chamber (1:9, BD Bioscience). Medium supplementing with 10% FBS was added in the lower chamber as a chemoattractant. Cells invaded to the basal side of the 8-μm membrane were then fixed with 4% paraformaldehyde for 15 min after incubation at 37 °C with 5% CO_2_ for 48 h. The morphology of the cells was stained with 0.1% crystal violet and the number of the cells was counted by a microscope (Nikon Microphot-FX, Japan) in five uniformly distributed fields.

### Western blot analysis

Cells were washed with ice-cold PBS and then lysed on ice in Western and IP cell protein lysis buffer(P0013, Beyotime) supplemented with 1% (*v*/v) protease inhibitor cocktail and phenylmethanesulfonyl fluoride (selleckchem, Houston, USA). The lysates were centrifuged at 12,000×g for 10 min at 4 °C. Protein concentrations were determined using a BCA Protein Assay Kit (Beyotime) and samples were then denatured by boiling. Total protein (25 μg /lane) was resolved using sodium dodecyl sulfate-polyacrylamide gel electropheresis (SDS-PAGE) and transferred onto a polyvinylidene fluoride (PVDF) membrane using a wet transfer system (Bio-Rad, USA) at 70 V and 4 °C. For immunoblotting, the PVDF membrane was incubated with Tris-buffered saline containing 0.1% (v/v) Tween-20 (TBS-T) and 5% (*w*/*v*) non-fat milk for 1 h, followed by incubation with the primary antibody overnight at 4 °C. Horseradish peroxidase (HRP)-conjugated IgG was used as the secondary antibody and membranes were incubated for 2 h. Afterwards, reactive protein was detected using an enhanced chemiluminescence (ECL) kit (Beyotime). The results were recorded using the MicroChemi Bio-Imaging System (DNR Bio-Imaging Systems Ltd., Jerusalem, Israel) and Quantity One version 4.5.0 software (Bio-Rad, Hercules, CA, USA).The primary antibodies used for this study were as follows: rabbit anti-TGF-β1 polyclonal antibody (1:1000; Cat no. 18978–1-AP; Proteintech Group), and mouse anti-tubulin monoclonal antibody (1:2000; Cat no. 0098; Cwbiotech, Beijing, China). The secondary antibodies included goat anti-rabbit IgG serum (1:40000; Zhongshan Golden Bridge, Beijing, China) and goat anti-mouse IgG serum (1:40000; Zhongshan Golden Bridge).

### Lentivirus construction and infection

pri-miR-663a sequence was cloned into pLenti-puro vector (Invitrogen, Carlsbad, USA) to form pLenti-miR-663a. pLenti-miR-663a or pLenti negative control (NC) vector was transfected into 293 T. Viral particles were collected 48 and 72 h later, centrifuged them together at 4000 r.p.m. for 5 min at 4 °C, then filtered with 0.45 μm filter. Huh-7 cells were infected with the viral particles at MOI of 20 and was then selected for 10 days with 0.8 μg /mL puromycin.

### Tumorigenicity assay in nude mice

Huh-7 cells that stably expressed the miR-663a or negative control (Huh-7-pLenti-miR-663a or Huh-7-pLenti-NC) were subcutaneously injected into five-week-old male BALB/c nude mice (Vital River, Beijing, China) at 5 × 10^6^ cells/site on right flank (re-suspended in 100 μl DMEM medium), respectively. Tumor growth was examined every 3 days for 4 weeks. Tumor volume was monitored by measuring in two directions with vernier calipers and formulated as tumor volume (V) = 1/2× length × width^2^. The trial was terminated and the mice were killed by sevoflurane inhalation when one mouse in any cohort had to be sacrificed for tumor burden according to the relevant regulations.

### Immunohistochemical (IHC)

TGF-β1 expression was determined by immunohistochemistry on paraffin­embedded tissue sample. Briefly, paraffin sections (3 μm) from tissue samples were deparaffinized in xylene and rehydrated in descending ethanol series according to standard protocol, and underwent antigen retrieval by microwave boiling in 10 mM citrate buffer for 2 min. Subsequently, sections were blocked with hydrogen peroxide for 10 min to quench endogenous peroxidase activity. The sections were incubated with normal goat serum to block non­specific binding and were then incubated overnight at 4 °C with rabbit anti-TGF-β1 polyclonal antibody (1:100; ABGEN Inc. San Diego, CA, USA) in a humidified chamber. For the negative control, normal goat serum was used to replace anti-TGF-β1 antibody. After washing, the sections were incubated with biotinylated goat anti-rabbit antibody conjugated with horseradish peroxidase (Maixin Inc. Fuzhou, China) at room temperature for 10 min as protocol. The sections were then visualized with DAB, counterstained with 10% Mayer’s hematoxylin, mounted in neutral gum, and analyzed using a bright field microscope.

### Statistical analysis

Results were presented as mean ± standard deviations (SD) from a minimum of three replicates. GraphPad Prism 5 (San Diego, CA, USA) was used for all statistical analyses in this study. The difference in miR-663a expression between HCC tissues and tissues adjacent to HCC were examined using paired sample t-test. Unless otherwise noted, the differences was analyzed using independent Student’s t-test for unpaired two-group comparisons and one-way ANOVA followed by the Student-Newman-Keuls test for multiple-group comparisons. The chi-square test and nonparametric Mann-Whitney U test were used to analyze the association between miR-663a expression level and various clinicopathologic characteristics. Bivariate correlation between miR-663a and TGF-β1 mRNA expression was assessed by linear regression model and pearson correlation coefficient. Statistical differences were considered statistically significant when *P* < 0.05 (2-sided significance testing).

## Results

### miR-663a is downregulated in HCC

**Fig. 1:**
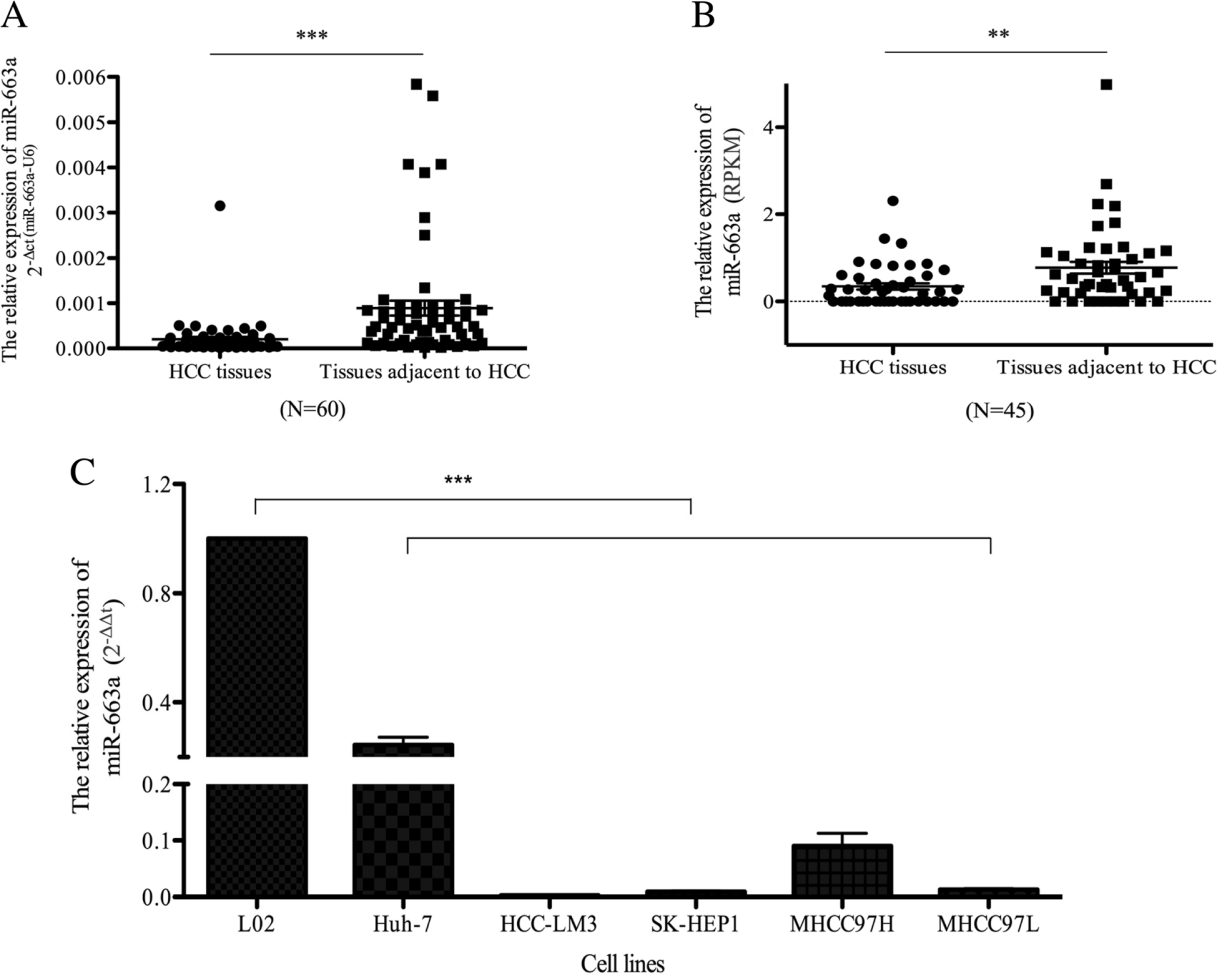
miR-663a was low-expressed in HCC tissues and cell lines. **a** qRT- PCR analysis of miR-663a expression in 60 pairs HCC and their adjacent nontumor liver tissues. The relative expression of miR-663a was normalized to U6 snRNA. **b** Analysis of microarray data from the Cancer Genome Atlas project database (TCGA) database. **c** qRT- PCR analysis of miR-663a expression in HCC cell lines (Huh-7, HCC-LM3, SK-HEP1, MHCC97H and MHCC97L) and normal immortalized liver cell line (L02). Data are presented as mean ± SD (*n* = 3). **p < 0.01, ***p < 0.001

To explore the potential expression pattern of miR-663a in HCC tissues, 60 pairs of HCC tissues and their adjacent tissues were analyzed using qRT- PCR. The results showed that miR-663a was significantly lower in the HCC tissues than those in matched non-tumorous tissues (Fig. 1a). To further confirm the expression of miR-663a in HCC, Cancer Genome Atlas project database (TCGA, including 45 paired HCC tissues and tissues adjacent to HCC) was used to exploit the expression levels of miR-663a (reads per kilobase per million mapped reads-RPKM). As shown in Fig. 1b, the expression of miR-663a was obviously downregulated in HCC from the data of TCGA (*P* < 0.01). Consistent with the results we observed in HCC tissues, the average level of expression of miR-663a was significantly lower in a panel of five HCC cell lines than that in L02, a human immortalized liver cell line (Fig. 1c). Taken together, all these results indicated that miR-663a was downregulated in HCC.

### miR-663a expression is associated with clinicopathological features of HCC patients

The correlation between miR-663a expression and various clinicopathological characteristics of HCC tissues was shown in Table 2. miR-663a expression was positively associated with tumor size (*P* = 0.036) and tumor number (*P* = 0.002), respectively. However, it was no correlations with age, gender, HBsAg status, cirrhosis, preoperative serum AFP, PVTT, histological differentiation and TNM stage.

**Table 2:** Clinicopathological features of miR-663a expression in hepatocellular carcinoma patients^a^

Clinicopathological features	No. of cases (*N* = 186)	miR-663a expression		*P-value*
		High (n %)	Low (n %)	
Age (year)				
≥50	156	80(86.0%)	76(81.7%)	0.550
< 50	30	13(14.0%)	17(18.3%)	
Gender				
Male	160	78(83.9%)	82(88.2%)	0.527
Female	26	15(16.1%)	11 (11.8%)	
HBsAg status				
Positive (+)	132	63(67.7%)	69(74.2%)	0.419
Negative (−)	54	30(32.3%)	24(25.8%)	
Cirrhosis				
YES	131	64(68.8%)	67(72%)	0.748
NO	55	29(31.2%)	26(28%)	
AFP (ng/ml)				
≥7	109	57(61.3%)	52(55.9%)	0.552
< 7	77	36(38.7%)	41(44.1%)	
PVTT				
YES	37	15(16.1%)	22(23.7%)	0.270
NO	149	78(83.9%)	71(76.3%)	
Tumor size(cm)				
≥5	111	48(51.6%)	63(67.7)	0.036*
< 5	75	45(48.4%)	30(32.3)	
Tumor number				
Single	38	28(30.1%)	10(10.8%)	0.002*
Multiple	148	65(69.9%)	83(89.2%)	
Tumor differentiation				
Well-moderate	151	72(86.0%)	79(84.9%)	0.129
Poor	35	21(14.0%)	14(15.1%)	
TNM stage (Edition 7th)				
I	118	62(66.7%)	56(60.2%)	0.341
II	36	17(18.3%)	19(20.4%)	
III	32	14(15.0%)	18(19.4%)	

^a^*AFP* α-fetoprotein, *HBsAg* Hepatitis B surface antigen, *TNM* Tumor-node-metastasis, *PVTT* Portal vein tumor thrombus

*P < 0.05

### Ectopic expression of miR-663a inhibits HCC cell proliferation

**Fig. 2:**
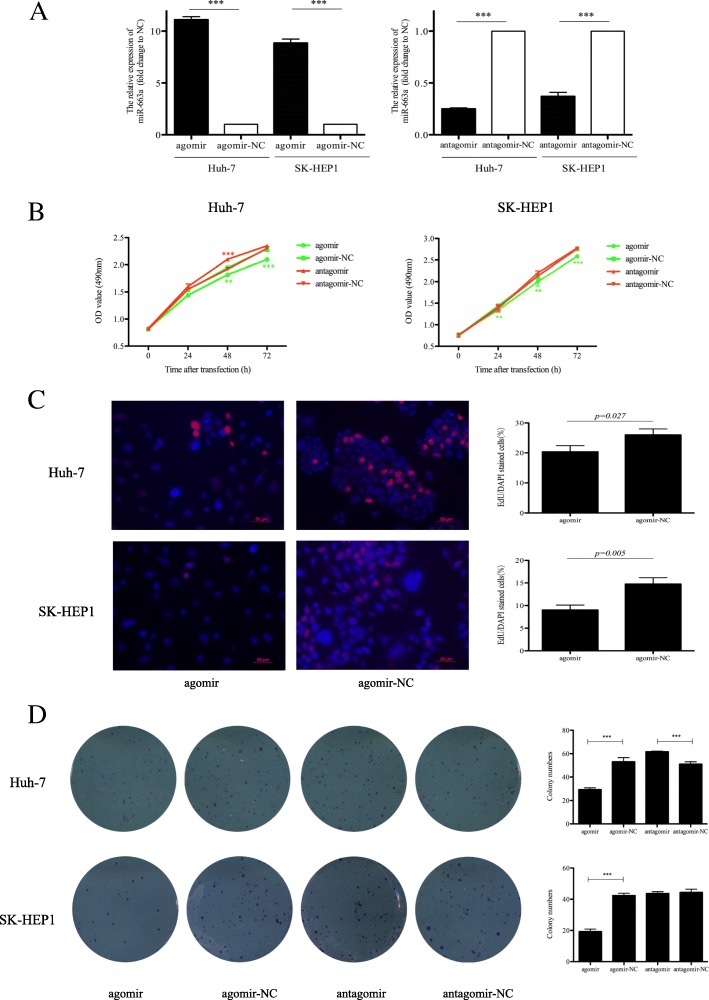
miR-663a inhibited HCC cell proliferation abilities. **a** Level of miR-663a in Huh-7 and SK-HEP1 cells transfected with miR-663a agomiR, antagomiR and respective control as assessed by qRT-PCR. The expression of miR-663a was normalized against U6 snRNA. **b** The MTS assay analysis was used to evaluate the proliferation of HCC cells transfected with miR-663a agomiR, antagomiR and respective control. **c** The EdU assay analysis was used to evaluate the proliferation of HCC cells transfected with miR-663a agomiR and control. **d** The colony formation assay analysis was used to evaluate the proliferation of HCC cells transfected with miR-663a agomiR, antagomiR and respective control. Representative fields are shown (400×). Data are presented as mean ± SD (*n* = 3). **p < 0.01, ***p < 0.001

To establish miR-663a overexpressing and knockdown cells, we upregulated or downregulated the expression of miR-663a in Huh-7 and SK-HEP1 cells by the transfecting miR-663a agomiR or antagomiR, respectively. The expression of miR-663a was confirmed to be significantly increased or decreased in the transfected cells by qRT–PCR (Fig. 2a). We found that overexpressed miR-663a reduced, whereas downregulated miR-663a promoted Huh-7 cell proliferation in MTS, EdU, and colony formation assays. In SK-HEP1 cells, miR-663a overexpression led to a significant reduction in MTS, EdU, and colony formation assays (Fig. 2b–d). These results proved that miR-663a inhibited the cell proliferation in HCC.

### Restoration of miR-663a represses the invasion of HCC cells

**Fig. 3:**
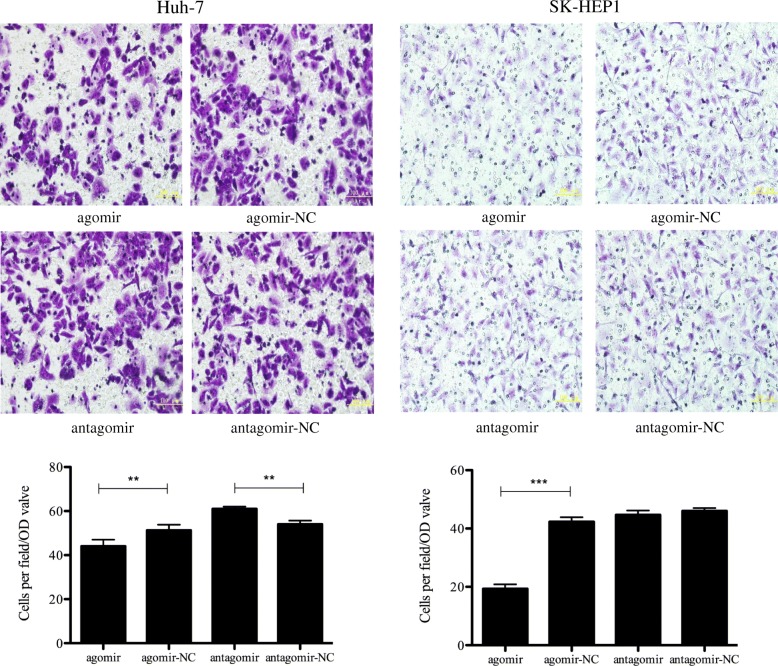
Effect of miR-663a on invasion in HCC. Transwell assay analysis was used to evaluate the invasive capacity of HCC cells transfected with miR-663a agomiR, antagomiR and respective control. Representative fields are shown (400×). Data are presented as mean ± SD (*n* = 3). **p < 0.01, ***p < 0.001

Transwell chamber assay was performed to investigate the function of miR-663a in HCC invasion. We found that the increased expression of miR-663a in Huh-7 and SK-HEP1 cells could significantly inhibit cell invasion abilities. Remarkably, the number of Huh-7 cells transfected with antagomiR passing through the matrigel was significantly higher than that of its control (Fig. 3). Collectively, our results suggested that miR-663a played a suppressive role in HCC invasion.

### TGF-β1 is a direct target of miR-663a in HCC

**Fig. 4:**
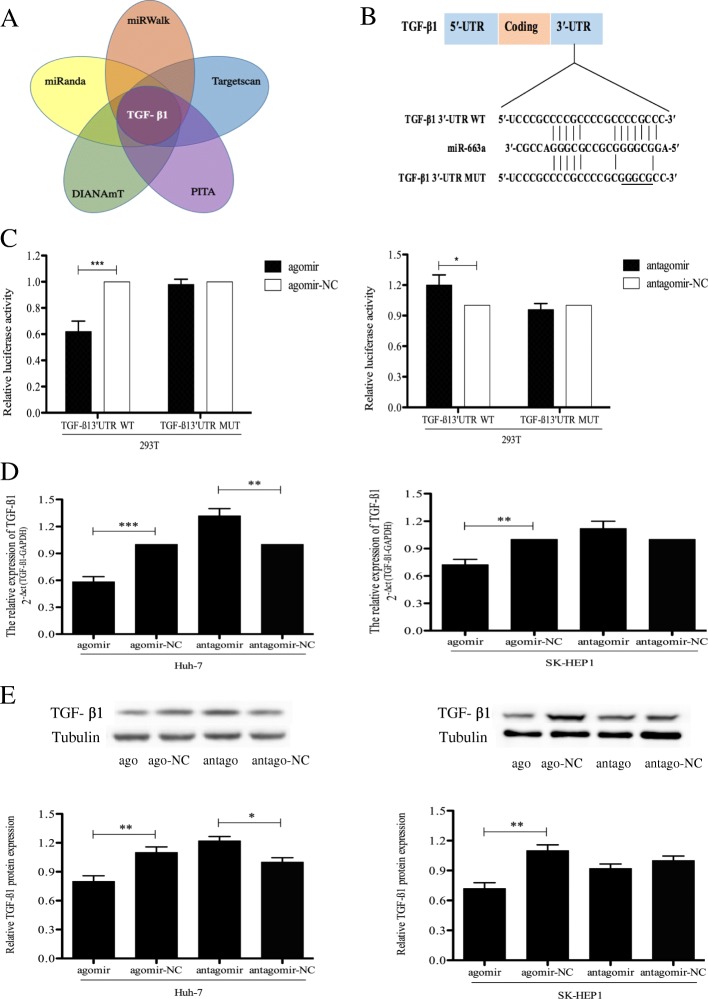
miR-663a directly targeted TGF-β1. **a** The overlap of TGF-β1 by bioinformatics prediction between miRWalk, TargetScan, PITA, DIANAmT and miRanda programs. **b** Diagram of the putative binding site of miR-663a on the 3′UTR of TGF-β1 predicted by Targetsan. The mutant sequences of 3′-UTR of TGF-β1 used in luciferase reporter is shown in transverse line. **c** Relative expression of luciferase reporters with wild type TGF-β1 3′-UTR or mutant TGF-β1 3′-UTR after co-transfection with miR-663a agomir, antagomir or respective control in 293 T. **d** qRT-PCR analysis of TGF-β1 mRNA expression in the HCC cells transfected by either miR-663a agomir, antagomir or respective control. The expression of TGF-β1 was normalized against GAPDH. **e** Western blot analysis of TGF-β1 protein expression in the HCC cells transfected by either miR-663a agomir, antagomir or respective control. The expression of TGF-β1 was normalized against Tubulin. Data are presented as mean ± SD (*n* = 3). *p < 0.05, **p < 0.01 and ***p < 0.001

To explore the potential molecular mechanism of miR-663a inhibiting invasion of HCC, miRwalk, a comprehensive database on miRNAs with eight established programs (miRanda, miRDB, miRWalk, TargetScan, RNA22, DIANAmT, PICTAR, and PITA), was used to predict the putative target genes of miR-663a [24]. TGF-β1 was one of the 841 putative miRNA sites that were predicted to binding miR-663a by miRWalk, TargetScan, PITA, DIANAmT, and miRanda programs (Fig. 4a and Additional file 1) and related to the role in HCC metastasis according to the relevant reports [25, 26]. We then identified potential binding sites for miR-663a at the 3′UTR of TGF-β1 mRNAs using TargetScan and a dual-luciferase reporter system to confirm whether TGF-β1 was a direct target of miR-663a. miR-663a was effective to reduce the luciferase activity of 293 T cells expressing wild-type TGF-β1 3′-UTR but not cells expressing mutant-type TGF-β1 3′-UTR in 293 T cells (Fig. 4b and c). As predicted, overexpression of miR-663a in Huh-7 and SK-HEP1 cells decreased the expression of TGF-β1 at both the mRNA and protein levels, whereas miR-663a inhibitor increased TGF-β1 expression in Huh7 cells (Fig. 4d and e). Taken together, our results revealed that TGF-β1 was a direct target of miR-663a.

### Transwell chamber assay wasmiR-663a impairs TGF-β1-promoted HCC cell invasion

**Fig. 5:**
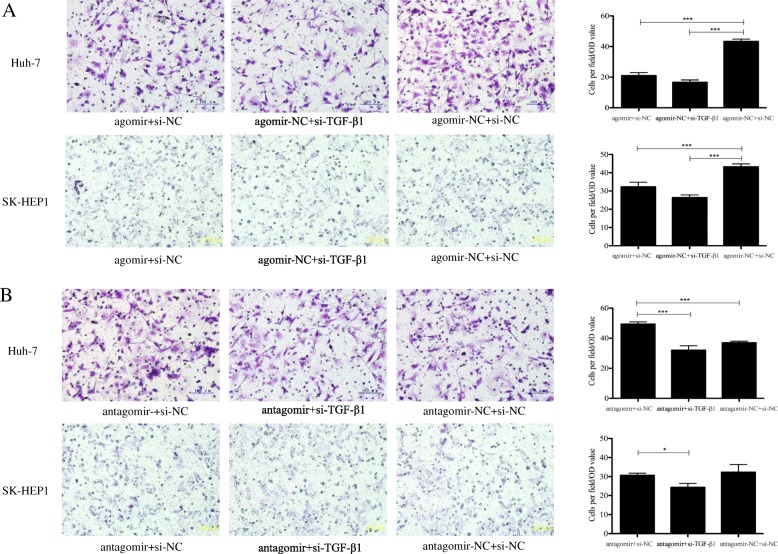
TGF-β1 was a functional target of miR-663a. **a** Downregulation of TGF-β1 expressions inhibited the invasion of HCC cells, which was similar to those induced by miR-663a agomir. **b** miR-663a antagomir-induced cell invasion was reversed by the knockdown of TGF-β1. Representative fields are shown (400×). Data are presented as mean ± SD (*n* = 3). *p < 0.05, ***p < 0.001

To confirm whether miR-663a-dependent anti-invasive behaviors of HCC cells are mediated by TGF-β1, the expression of TGF-β1 was knocked down in the corresponding Huh-7 and SK-HEP1 cells using specific small interfering RNA (siRNA). The results of transwell assay revealed that silence the expression of TGF-β1 significantly inhibited the invasion of Huh-7 and SK-HEP1 cells, which were similar to the effect of miR-663a overexpression (Fig. 5a). We further assessed whether knockdown of TGF-β1 could reverse the invasion promotive effect of miR-663a inhibitor. As shown in Fig. 5b, TGF-β1 downregulation significantly attenuated the abilities of cell invasion promoted by miR-663a antagomir in Huh7 cells. These results demonstrated that miR-663a inhibited the invasion of HCC cells by targeting TGF-β1.

### miR-663a inhibits the tumorigenesis of HCC cells in nude mice

**Fig. 6:**
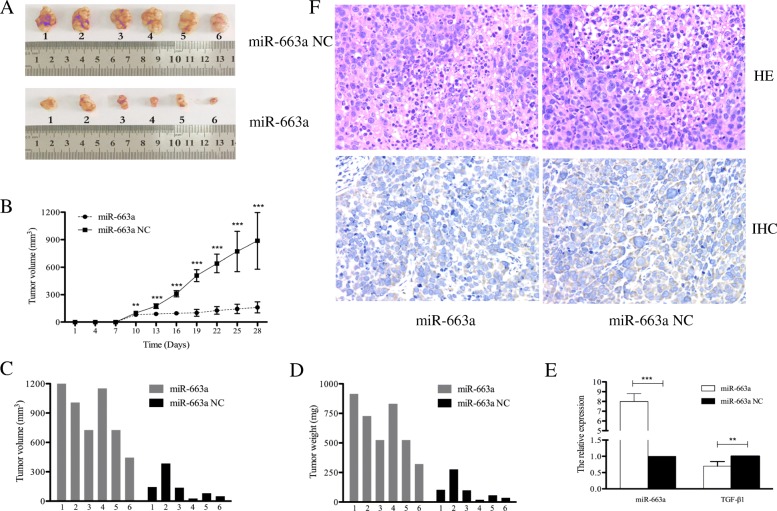
miR-663a suppressed tumorigenesis in vivo. **a** Tumor formation in nude mice 4 weeks after injection with Huh-7- pLenti-miR-663a and Huh-7- pLenti-NC. **b** Growth curve of Huh-7- pLenti-miR-663a and Huh-7- pLenti-NC-formed tumors. Volumes of the tumors were measured every 3 days. (Data are presented as mean ± SD (*n* = 6)). **c** Volumes of the Huh-7- pLenti-miR-663a and Huh-7- pLenti-NC-formed tumors 4 weeks after the initial injection. **d** Weights of the Huh-7- pLenti-miR-663a and Huh-7- pLenti-NC-formed tumors 4 weeks after the initial injection. **e** The expression of miR-663a and TGF-β1 was detected by qRT–PCR in the mouse tumor tissues induced by Huh-7- pLenti-miR-663a and Huh-7- pLenti-NC. (Data are presented as mean ± SD (*n* = 3)). **f** The expression of TGF-β1 in tumor tissues was measured by immunohistochemistry and HE staining. Representative fields are shown (400×). **p < 0.01, ***p < 0.001

Since miR-663a regulates the growth of HCC cells in vitro, we further assessed its effect in vivo. Huh-7 cells stably expressing miR-663a or miR-663a NC were subcutaneously inoculated into nude mice (*n* = 6), respectively. The size of tumors in the mice was measured using a caliper every 3 days. Consistent with the results in vitro, the tumor volume was significantly decreased in the group injected with the miR-663a compared with the negative control group (Fig. 6a–c). The tumors were then extracted after implantation for 4 weeks, and tumor weight of the miR-663a group was also significantly lower compared to the control group with the same relationship (Fig. 6d). The miR-663a and TGF-β1 expression in xenograft tumors was then determined using qRT-PCR. Results showed that miR-663a expression was upregulated. However, TGF-β1 mRNA levels were significantly lower in the xenograft tumors from the miR-663a group than in the control group (Fig. 6e). Consistently, downregulation of TGF-β1 was observed in the xenograft tumors from the miR-663a group relative to the control group according to the results of immunohistochemical analysis. These data indicated that overexpression of miR-663a may inhibit HCC tumorigenesis by blocking TGF-β1 expression.

### miR-663a regulates TGF-β1 expression in clinical HCC specimens

**Fig. 7:**
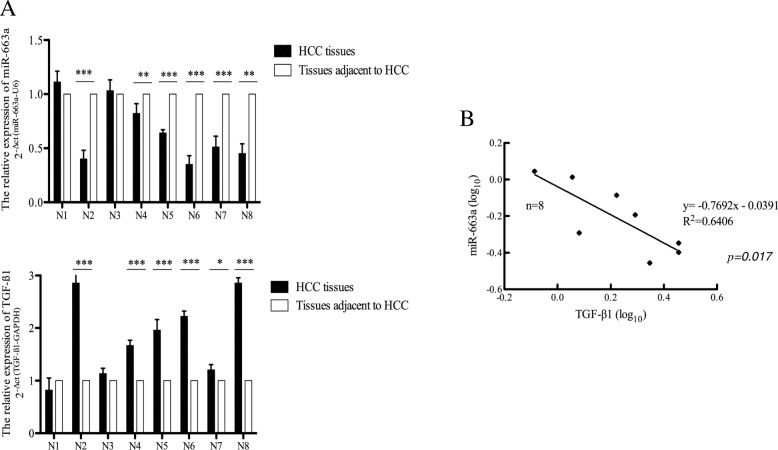
Decline of miR-663a related with the up-regulation of TGF-β1 in HCC tissues. (**a**) Expression of miR-663a and TGF-β1 were detected by the qRT-PCR in 8 pairs of HCC samples. miR-663a was decreased in 6 of 8 HCC samples. N, Patient number. (**b**) The correlation analysis between miR-663a and TGF-β1 in HCC tissues. Data are presented as mean ± SD (n = 3). *p < 0.05, **p < 0.01 and ***p < 0.001

To further investigate whether miR-663a is involved in regulation of TGF-β1 in HCC tissue specimens, the expression of miR-663a and TGF-β1 were analyzed by qRT-PCR in 8 pairs of clinical HCC samples. Compared to the tissues adjacent to HCC, the expression of miR-663a was reduced in 6 of the 8 HCC tissues. In accordance with miR-663a decline (median, 0.58-fold; range, 0.35- to 1.11-fold), the expression of TGF-β1 (median, 1.82-fold; range, 0.82- to 2.86-fold), was increased in all 6 HCC tissues (Fig. 7a). Correlation analysis indicated that miR-663a expression was reduced, along with TGF-β1 overexpression in these 8 HCC specimens (*R*^2^ = 0.6406, *P* = 0.017, Fig. 7b).

## Discussion

HCC is a highly aggressive malignant neoplasm found in patients all over the world. Surgical resection, liver transplantation, and local ablation therapy are the curative strategies available during the early stages of HCC. However, the treatment is based merely on TACE, biotherapy, and supportive and palliative care in the majority of patients diagnosed at the advanced stage [27]. In general, therapeutic intervention for HCC is not fully explicit and can be partially ineffective due to the insufficient recognition of biological and genetic heterogeneities of the tumor. Although numerous alterations in the genome, transcriptome, proteome, and metabolome of HCC have been identified, the molecular mechanism underlying HCC remains to be investigated.

A fairly large number of miRNAs have been found to be dysregulated in HCC and affect tumor growth, migration, invasion, and drug resistance by regulating the coding and non-coding sequences of the genes [28, 29]. miR-663a has been proven to be abnormal in many solid tumors. However, the role of miR-663a in tumorigenesis is very complicated and may be organ-specific. miR-663a consistently suppresses tumorigenic features in breast tumors [14, 30], colon cancer [31, 32] pancreatic cancer [33–35], and glioblastoma [15, 16, 36], but increases carcinogenic characteristics in prostate cancer [21, 37, 38] and nasopharyngeal carcinoma [19, 39, 40]. Currently, studies of miR-663a on HCC have yielded bidirectional results. Huang Weizhen et al. found that miR-663a was significantly downregulated in HCC tissues when compared with the adjacent non-tumor tissues from the GSE21362 and TCGA databases. miR-663a inhibited HCC cell proliferation and metastasis by directly targeting HMGA2, which suggested that miR-663a may serve as an anticancer target for HCC [22]. Wang Guanyu et al. demonstrated that miR-663a was specifically downregulated and involved in the development of HBV-related HCC using microarray analyses [41]. However, Huang Yawei et al. reported that the downregulation of miR-663a suppressed HCC cell proliferation and promoted apoptosis under endoplasmic reticulum stress by directly targeting TGF-β1, indicating that miR-663a acts as an oncogene in HCC under some circumstances [23].

TGF-β1 is an important cytokine since it participates in a series of critical biological processes such as cell proliferation, apoptosis, invasion, and migration. TGF-β1 has a complicated role in tumor cells; it suppresses tumor formation during the initial stages of tumor development and then promotes metastasis during the later stages [42]. There is a comprehensive and paradoxical relationship between miR-663a and TGF-β1 in the current study. Li Qizhuang et al. showed that miR-663a reduced the proliferation, migration, and invasion of glioblastoma cells by directly suppressing the expression of TGF-β1 and of downstream MMP2 and E-cadherin [36]. Quan Hong et al. indicated that miR-663a inhibited endothelial cell migration in a PTEN-dependent manner by regulating TGF-β1. Mody et al. observed that miR-663a directly targeted TGF-β1 and suppressed EMT and metastasis in pancreatic cancer [33]. However, Liu Zhiyong et al. demonstrated that miR-663a contributed to lung cancer cell proliferation by regulating TGF-β1 [20]. Thus, it would be meaningful to determine whether the impact of miR-663a on TGF-β1 may vary according to the tumor histological type, stage, and microenvironment.

Two basic characteristics, metastasis and proliferation, have been identified as the malignant biological determinants of cancer, including HCC [43]. In this study, we have confirmed that miR-663a was visibly decreased in HCC tumor tissues and cells. We also observed that the expression of miR-663a was significantly negatively related to the tumor size and number. In addition, we demonstrated that miR-663a could suppress the proliferation and invasion of HCC cells in vitro and inhibit tumor tumorigenesis in vivo, indicating the antineoplastic role of miR-663a in the occurrence and development of HCC. Then, the molecular mechanism of miR-663a in the progression of HCC was investigated. miR-663a could target TGF-β1 directly, which was consistent with the results of other studies. Our study showed that miR-663a inhibited the invasion of HCC via the decreased expression of TGF-β1. It is the first work to prove the downregulation of TGF-β1 by miR-663a of HCC xenografts in nude mice. Besides, the expression of miR-663a and the inversely correlated with the expression of TGF-β1 were also detected from the clinical HCC specimens. The above results indicate that miR-663a might affect the biological behavior of HCC by regulating TGF-β1.

The development of miR-663a as a potential therapeutic agent may have some limitations. For instance, miR-663a exerts some nonspecific actions, such as decreasing the expression of certain potential tumor suppressor gene (i.e., P21) and reducing the expression of certain possible oncogenes (e.g., HMGA2, eEF1A2, and PIK3CD). miR-663a is involved in the biological behavior of cell differentiation, inflammation, autoimmune diseases, and cancer, which could cause off-target effects and untoward effects unexpectedly. Further, the delivery of miR-663a is limited by many barriers. Virus-based vectors are limited by biosafety, targeting specificity, and immune responses, and non-virus-based carriers are challenged by nuclease degradation, renal clearance, phagocytic immune cells, low tissue/cell penetration, and toxicity [44]. Concentrations of miR-663a in fundamental research may differ by several orders of magnitude. The modification and optimization of miR-663a should be undertaken to reduce the effective dose, and the minimum effective dose of miR-663a should be determined by the dose–effect relationship in the preclinical study.

## Conclusion

Our study enriches the scope of miR-663a regulation in HCC, which may deepen our understanding of HCC tumor biology and motivate the development of miRNA-based antitumor therapies.

## Additional file

Additional file 1: The putative miR-663a binding sites. The 841 putative miRNA sites that were predicted to binding miR-663a by miRWalk, TargetScan, PITA, DIANAmT, and miRanda programs. (XLSX 64 kb)

## Abbreviations

3′UTR: 3′ untranslated region
AFP: α-fetoprotein
AJCC: American joint committee on cancer
EdU: 5-ethynyl-2′-deoxyuridine
eEF1A2: Eukaryotic translation elongation factor 1 alpha 2
EMT: Epithelial mesenchymal transition
ER: Endoplasmic reticulum
HBsAg: Hepatitis B surface antigen
HCC: Hepatocellular carcinoma
HMGA2: High-mobility group AT-hook 2
miR-663a: MicroRNA-663a
MMP2: Matrix metallopeptidase 2
MTS: 3-(4,5-dimethylthiazol-2-yl)-5-(3-carboxymethoxyphenyl)-2-(4-sulfophenyl)-2H-tetrazolium, inner salt
MUT: Mutant type
PIK3CD: Phosphatidylinositol 4,5-bisphosphate 3-kinase catalytic subunit delta
PTEN: Phosphatase and tensin homolog deleted on chromosome ten
PVTT: Portal vein tumor thrombus
qRT-PCR: Quantitative real time polymerase chain reaction
TACE: Transarterial chemoembolization
TCGA: The Cancer Genome Atlas
TGF-β1: Transforming growth factor beta 1
TNM: Tumor-node-metastasis
WT: Wild type
